# Matricellular protein tenascin C: Implications in glioma progression, gliomagenesis, and treatment

**DOI:** 10.3389/fonc.2022.971462

**Published:** 2022-08-12

**Authors:** Zaixiang Fu, Ganggui Zhu, Chao Luo, Zihang Chen, Zhangqi Dou, Yike Chen, Chen Zhong, Sheng Su, Fuyi Liu

**Affiliations:** ^1^ Department of Neurosurgery, Second Affiliated Hospital, School of Medicine, Zhejiang University, Hangzhou, China; ^2^ Department of Neurosurgery, Hangzhou First People’s Hospital, School of Medicine, Zhejiang University, Hangzhou, China; ^3^ Department of Neurosurgery, The Affiliated Hospital of Kunming University of Science and Technology, Kunming, China; ^4^ Department of Neurosurgery, The Fourth Affiliated Hospital, School of Medicine, Zhejiang University, Yiwu, China

**Keywords:** matricellular protein, tenascin C, glioma progression, neurogenesis, gliomagenesis, clinical significance

## Abstract

Matricellular proteins are nonstructural extracellular matrix components that are expressed at low levels in normal adult tissues and are upregulated during development or under pathological conditions. Tenascin C (TNC), a matricellular protein, is a hexameric and multimodular glycoprotein with different molecular forms that is produced by alternative splicing and post-translational modifications. Malignant gliomas are the most common and aggressive primary brain cancer of the central nervous system. Despite continued advances in multimodal therapy, the prognosis of gliomas remains poor. The main reasons for such poor outcomes are the heterogeneity and adaptability caused by the tumor microenvironment and glioma stem cells. It has been shown that TNC is present in the glioma microenvironment and glioma stem cell niches, and that it promotes malignant properties, such as neovascularization, proliferation, invasiveness, and immunomodulation. TNC is abundantly expressed in neural stem cell niches and plays a role in neurogenesis. Notably, there is increasing evidence showing that neural stem cells in the subventricular zone may be the cells of origin of gliomas. Here, we review the evidence regarding the role of TNC in glioma progression, propose a potential association between TNC and gliomagenesis, and summarize its clinical applications. Collectively, TNC is an appealing focus for advancing our understanding of gliomas.

## Introduction

The extracellular matrix (ECM) is a dynamic and complex meshwork consisting of various multidomain macromolecules that are continually synthesized and secreted by surrounding cells. Many ECM molecules, the basic structural proteins, provide a three-dimensional structural framework that ensures cell cohesion and facilitates formation of tissues and organs (1, 2). In addition to structural matrix molecules, such as collagen and laminin, various non-structural proteins are present in the ECM in specific situations. These secreted nonstructural ECM components, called matricellular proteins (MCPs), are rapidly turned over, rather than remaining as stable structural elements (2, 3). MCPs present in the brain ECM include tenascin C (TNC), thrombospondins (TSPs), secreted protein acidic and rich in cysteine family (SPARC) proteins, and periostin (3). They are characterized by low expression levels in healthy adult tissues, but have high expression levels during development and are promptly upregulated under pathological conditions. By binding to other matrix proteins, specific cell surface receptors, such as integrins, and soluble extracellular factors, including cytokines, growth factors, and proteases, MCPs can directly or indirectly modulate cellular morphology, regulate several cellular processes, such as proliferation, differentiation, migration, apoptosis, and survival, and induce tissue remodeling (3–5). TNC, as an MCP, is highly expressed in most solid cancers of the ECM. This has been associated with poor prognosis and is involved in many malignant biological behaviors (6, 7). In addition, TNC is present in several stem cell niches (8, 9), including neural stem cells (NSCs) and glioma stem cells (GSCs) (10–12). Therefore, understanding the role of TNC in cancers and stem cells can contribute to the development of new therapeutic avenues.

Gliomas, the most common primary intracranial tumors in adults, are grouped into four classes (grades I–IV), based on histological characteristics (13). The mean annual incidence is approximately six per 100,000 people worldwide (14). Although some advances in diagnosis and treatment have been made, the prognosis of patients with gliomas remains poor. In particular, for glioblastoma (GBM), the median survival time is approximately 15 months and the 5-year survival rate is only 5.8% (15–17). The standard regimen for primary GBM includes maximal surgical resection, followed by chemotherapy with temozolomide and radiation therapy, which is referred to as the STUPP protocol (18). Recently, many novel therapeutic options, such as immunotherapy (19, 20), targeted therapy (21, 22), and tumor-treating fields (TTFields) (23, 24) have been proposed, but the efficacy of these protocols is unsatisfactory (16). The main reasons for the poor outcomes of GBM patients are the disease’s heterogeneity and adaptability, which lead to resistance to treatment and tumor recurrence. The tumor microenvironment (TME) and cancer stem cells (CSCs) may account for the dynamic and heterogeneous characteristics of GBM (25, 26). Recently, substantial evidence has shown pleiotropic and important roles for TNC in the glioma TME (27, 28), as well as in NSC and GSC niches (10–12). Interestingly, many researchers have reported that GSCs may be derived from NSCs in the adult brain subventricular zone (SVZ) (29–31).

Here, in light of its potential significance, we review the matricellular protein TNC in the glioma ECM, and highlight the implications of TNC in glioma progression, tumorigenesis, and treatment.

## Structure of tenascin C

**Figure 1:**
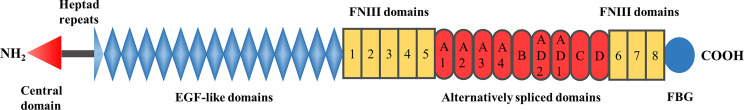
Schematic illustration of the domain structure of TNC.

TNC, a member of the tenascin family, was first identified in the 1980s. Other members of this family include tenascin-W, tenascin-X, and tenascin-R. Since the discovery of TNC independently and concurrently in several different laboratories, it is known under various names, such as glial/mesenchymal extracellular matrix protein (GMEM) (32), myotendinous antigen (33, 34), cytotactin (35), J1 220/200 (36), neuronectin (37), and hexabrachion (38). The TNC glycoprotein consists of six identical monomers that are disulfide-linked into a hexamer at their N-termini (38, 39). Each subunit is approximately 180–400 kDa in humans and is composed of four different parts **(** **Figure 1** **)**: an N-terminal cysteine-rich domain with highly conserved heptad repeats, 14.5 epidermal growth factor (EGF)-like repeats, eight constitutively expressed fibronectin type III (FNIII) domains, and a fibrinogen-like globe (FBG) at the C-terminus (28, 40, 41). Nine alternatively spliced domains (A1–A4, B, AD2, AD1, C, and D) are inserted between the 5th and 6th FNIII domain in the human gene (42), which can theoretically give rise to 512 possible TNC splice isoforms (43, 44). However, the number of alternatively spliced domains varies among species, with chickens, mice, and rats having six, six, and seven domains, respectively (43, 45, 46). These alternatively spliced repeats with unique interaction sites may not only offer novel binding abilities or susceptibilities to proteolytic cleavage, but may also disrupt the existing binding sites, resulting in the acquisition or loss of certain functions (43). In addition, post-translational modifications, such as glycosylation and citrullination, assembly into a fibrillar matrix, and proteolytic processing, further increase the complexity of the TNC structure and function by exposing hidden binding sites, covering exposed binding sites, or generating smaller soluble fragments (6, 43, 44). For example, glycosylated TNC is likely to regulate proliferation of NSCs (47), and the fragmented EGF-like domain has proapoptotic activity in smooth muscle cells, in contrast to intact TNC (48).

## Expression and regulation of tenascin C

TNC is a regulatory glycoprotein that exhibits different spatial and temporal distribution patterns throughout life. In general, during embryogenesis, TNC is highly expressed in neural ectodermal tissues and in some non-neural sites where high cell turnover, tissue remodeling, and epithelial–mesenchymal interactions occur (41, 43). In the embryonic developmental stage and shortly after birth, the expression level of TNC peaks and then decreases significantly with increasing age (49). In fact, this molecule is considered to be mainly secreted by immature and reactive astrocytes, radial glial progenitor cells, and oligodendrocyte precursor cells (OPCs) in the developing central nervous system (CNS) (50–52). In contrast, in the normal adult CNS, it is sparsely expressed and is confined to NSC niches, such as the SVZ and the hippocampus, where it is produced by astrocytes (53) and granule cells (54), respectively, as well as to the cerebellum, where it is produced by Golgi epithelial cells (55). However, TNC is actively re-expressed in the adult CNS in response to pathological conditions, including neuroinflammation (56), neurodegeneration (57, 58), trauma (59, 60), and tumorigenesis (27, 28, 61). For example, many experimental studies have indicated that TNC is expressed in the brain parenchyma (astrocytes, neurons, and brain capillary endothelial cells) and the walls of cerebral arteries (endothelial, smooth muscle, adventitial, and periarterial inflammatory cells) between 24 and 72 h after post-subarachnoid hemorrhage (SAH) (62, 63). As a key regulator of neuroinflammation, TNC is involved in early brain injury, including blood–brain barrier destruction (64), neuronal apoptosis (65), cerebral vasospasm (66), delayed cerebral ischemia (67), and chronic hydrocephalus (56, 62, 68). In addition, Xie et al. (69) reported that TNC plays a role in chronic neuroinflammation in Alzheimer’s disease, and that its deficiency could produce an anti-inflammatory pattern and reduce cerebral amyloid β load. More importantly, numerous studies have reported increased levels of TNC in multiple malignant solid tumors, with the highest concentrations found in gliomas (7, 27, 70). TNC expression is correlated with glioma grade, poor patient survival, and disease progression (71, 72).

In humans, *TNC* is located on chromosome 9q32–q34 (73). Upstream of its transcription start site, there is a region with high promoter activity that contains a TATA box (44, 74). Various specific transcription factors, intracellular regulators, and stimuli, including cytokines, growth factors, reactive oxygen species, hypoxia, and mechanical stress, can control *TNC* expression by directly or indirectly regulating the *TNC* promoter (74). For instance, homeobox even-skipped homolog protein-1 (Evx-1) stimulates *TNC* promoter activity by synergizing with transcription factors FOS and/or JUN, which target the AP1 site (75). Another homeobox transcription factor, orthodenticle homolog 2 (OTX2), binds to the *TNC* promoter with high affinity and represses the gene transcription (76). In addition, cyclic tensile strain has been shown to induce mRNA encoding TNC in fibroblasts in β1-integrin-mediated RHO/ROCK signaling (77, 78) and nuclear translocation of the transcriptional regulator megakaryocytic leukemia-1 (MKL-1) (79). After CNS injury, activated microglia and macrophages secrete basic fibroblast growth factor (bFGF) and transforming growth factor-β1 (TGF-β1), which can induce an increase in astrocyte tenascin production (80). Moreover, TNC was also considered a target gene of the transcription factor SOX4, which is overexpressed in many human malignancies, including glioma (81, 82).

**Figure 2:**
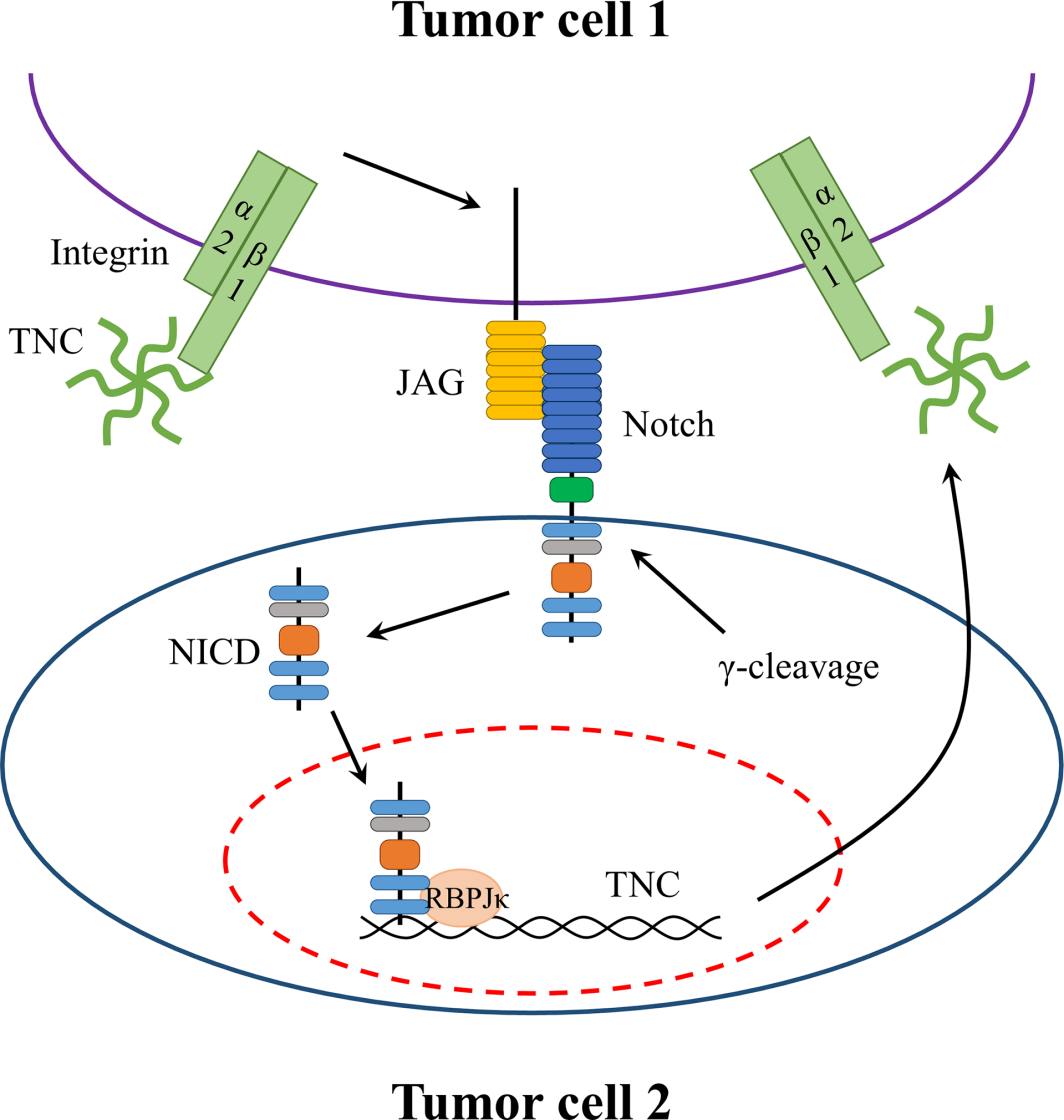
Potential positive feedback mechanism in gliomas between TNC expression and NOTCH pathway. TNC binds to integrin α2β1 on the glioma cell and upregulates JAG1 expression which interacts with its receptor NOTCH. The interaction results in the release of intracellular domain that translocates to the nucleus to affect NOTCH-dependent transcription by binding to RBPJκ. Moreover, the human TNC promoter contains an RBPJκ-responsive element. Thus, the activation of NOTCH signaling increases TNC expression.

Fibroblasts are a major source of the TNC deposited in the solid tumor stroma of the peripheral system, whereas tumor cells themselves rarely express TNC (70). Nevertheless, in gliomas, TNC is expressed by malignant tumor cells (83). The NOTCH signaling pathway plays a critical role in the regulation of TNC expression **(** **Figure 2** **)** (84). NOTCH is a large transmembrane receptor of the cell-binding ligands delta and jagged. After activation, ligand-dependent cleavage allows the release of its intracellular domain, which translocates to the nucleus and affects NOTCH-dependent transcription by binding to RBPJκ/CSL (85). A GBM tissue microarray revealed a significant association between RBPJκ and TNC levels, with the former being a NOTCH2 transcription co-factor (84). Additionally, the human *TNC* promoter contains an RBPJκ-responsive element (84). Sivasankaran et al. (84) proposed a mechanism for NOTCH/RBPJκ-mediated transactivation of *TNC* in GBM, consistent with the report by Ma et al. (86), in which TNC is upregulated in CD47-loss-of-function cells *via* a NOTCH-mediated mechanism. On the other hand, Sarkar et al. (87). found that TNC is a pivotal initiator of enhanced NOTCH signaling and promotes GSC growth through the TNC–α2β1–JAG1–NOTCH signaling axis. In breast cancer cells, Oskarsson et al. (88) reported that TNC accumulation enhanced the performance and function of the NOTCH pathway, which is vital for the adaptation of metastasis-initiating breast cancer cells. Taken together, evidence suggests that there may be positive feedback between TNC expression and the NOTCH pathway that ultimately increases the malignant biological behavior of gliomas.

## Interaction partners and receptors of tenascin C

As mentioned above, TNC is a hexameric extracellular glycoprotein and each monomer consists of four different domains. This highly complex structure gives TNC the capacity to interact with various binding partners or ligands, such as other ECM components, cell surface receptors, and soluble factors, which affect distinct signaling pathways (28, 44, 70, 89). The first and most studied ECM component that interacts with TNC is fibronectin (89), whose binding sites involve FNIII repeats (90, 91). Additionally, TNC can bind to other ECM proteins, such as periostin (92), perlecan (93), fibrillin-2 (94), aggrecan (95), and SPARC-related modular calcium-binding protein 1 (SMOC1) (96). These complex interactions between TNC and ECM proteins may contribute to changes in the matrix components and the biological properties of the TME. TNC can also bind to proteoglycans (PGs), such as receptor-like protein tyrosine phosphatase beta/zeta (RPTPβ/ζ) (97) and neurocan (98), two nervous tissue-specific chondroitin sulfate proteoglycans: glypican (99), syndecan-4 (100), and two heparan sulfate proteoglycans. These PGs are involved in tumor or stem cell adhesion and proliferation by interacting with multiple domains of TNC or peptides derived from TNC. In addition to PGs, TNC can act directly on cell surface receptors, particularly integrins. Yoshida et al. (70) reported that TNC plays an essential role in cancer cell biology as a ligand for integrins α2/7/8/9β1 and αvβ1/3/6. The EGF-like repeats of TNC are capable of promoting cell proliferation by binding directly to the EGF receptor and activating the ERK/MAPK signaling pathway (101), whereas the FBG domain of TNC is able to maintain the synthesis of proinflammatory cytokines *via* activation of Toll-like receptor 4 (TLR4) in a myeloid differentiation factor-88 (MyD88)-dependent manner (102). Furthermore, a large number of growth factors have been found to bind to the TNC FNIII 1–5 subdomain, including the platelet-derived growth factor (PDGF) family, TGF-β superfamily, FGF family, insulin-like growth factor-binding proteins (IGF-BPs), and neurotrophins (103). More recently, a few studies have suggested that TNC participates in recruiting and concentrating WNT ligands in acute kidney injury and whisker follicle stem cell niches, thereby potentiating WNT/β-catenin signaling, presumably due to the formation of a favorable microenvironment near the cell surface (104, 105). Finally, TNC can even bind to pathogens, such as human immunodeficiency virus, and neutralize viral activity (106). Taken together, the interaction partners of each TNC isoform are thought to be dependent on their domains, thus performing different functions in a context-dependent manner. Notably, TNC harbors some cryptic functional sites within its molecular structure, which are released through proteolytic cleavage by matrix metalloproteinases (MMPs) and a disintegrin and metalloproteinase (ADAM) family. The TNC-derived peptide fragments, such as FNIII A2, contain bioactive sites and may play a different and even opposite role than that of the parental TNC molecule (107).

## Tenascin C in glioma

TNC is abundantly expressed in a variety of tumors, including breast cancer, colon adenocarcinoma, prostatic adenocarcinoma, and lung carcinoma, but particularly in gliomas (7, 27, 28, 70). In general, TNC is mainly present in the glioma perivascular and intercellular spaces and less abundantly in the cells (108, 109), and its expression level increases with glioma grade (71, 109). Interestingly, the concentration of TNC in the cerebrospinal fluid and cyst fluid also seems to reflect the tumor grade (110, 111). Among patients with GBM, those who had TNC immunopositivity survived for a significantly shorter period than those in whom TNC expression was absent (71). Thus, TNC in gliomas can be identified as a predictor of poor prognosis and disease progression (71, 72). Unlike most other tumors, malignant glioma cells are the main source of TNC (112). TNC can also promote many malignant biological behaviors of glioma, such as neovascularization, proliferation, adhesion or migration, and immunomodulation (27, 28).

### Tenascin C and neovascularization

**Figure 3:**
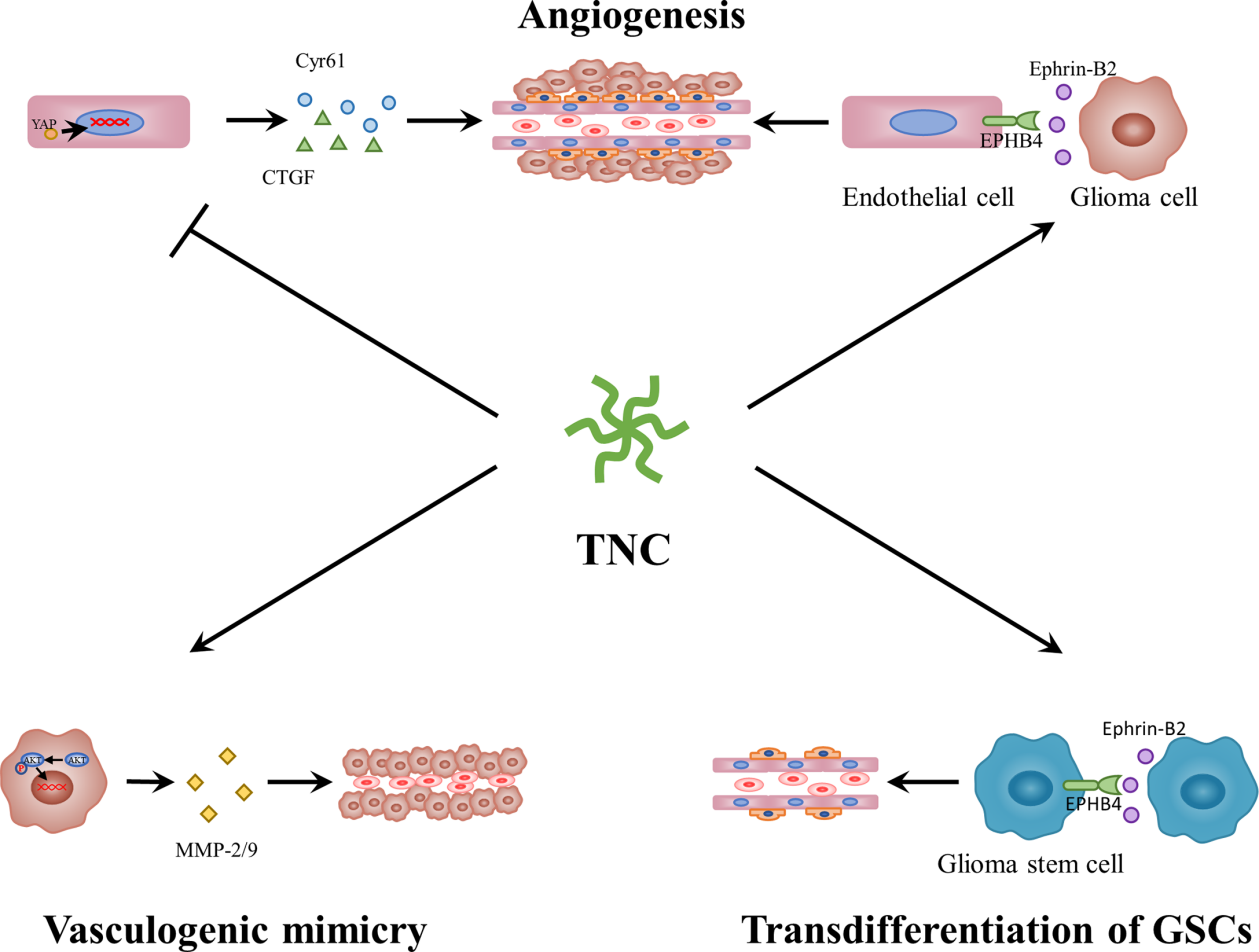
The roles of TNC in glioma neovascularization. On the one hand, TNC blocks YAP signaling and endothelial cell behavior through direct contact. On the other hand, TNC induces ephrin-B2 and a pro-angiogenic secretome in glioblastoma cells. In addition, TNC activates AKT/MMP2/MMP9 axis and further promotes vasculogenic mimicry in glioma. Moreover, TNC-ephrinB2-ephB4 signaling pathway supports GSCs differentiation into endothelial cells. YAP, Yes-associated protein; MMP, matrix metalloproteinase.

Gliomas are characterized by a high degree of vascularization. These blood vessels are necessary for tumor growth, as they are involved in providing nutrients and removing metabolic waste. Over the past few years, some studies have demonstrated that TNC is mainly found in the perivascular niche of gliomas (72, 108), particularly in hyperplastic blood vessels (113, 114). The microvessel endothelial cells (114), pericytes (115) and smooth muscle cells (116) all have the capacity to synthesize and release TN/TNC. In GBM, perivascular TNC is related to microvascular density and vascular endothelial growth factor (VEGF) expression (108). In addition, in melanoma, TNC can regulate the expression of VEGF and affect angiogenesis in tumors (117). Proteome and immunohistochemical comparisons between tissues with physiological angiogenesis and GBM angiogenesis indicated aberrant upregulation of TNC in the latter (118). Therefore, TNC may play an important role in glioma neovascularization **(** **Figure 3** **)**. Recently, Rosińska et al. (119) reported that gliomas deploy diverse neovascularization means to meet a dedicated blood supply, including co-option, angiogenesis, vasculogenesis, vascular mimicry (VM), and transdifferentiation of GSCs. First, TNC is linked to angiogenesis. Zagzag et al. (120) discovered that TNC acts as a permissive substrate that promotes microvascular cell migration *in vitro* by triggering focal adhesion kinase (FAK) phosphorylation in endothelial cells. In *TNC*-knockdown GBM-neurosphere intracranial xenografts, tumor blood vessel density was lower, while the lumen was enlarged as compared to the control (121). It is important to mention that Rupp et al. (122) demonstrated the dual angiogenic effects of TNC in GBM **(** **Figure 3** **)**. The direct contact between endothelial cells and TNC represses actin polymerization, impairs YAP signaling, and downregulates pro-angiogenic factors, consequently negatively influencing endothelial cell proliferation, survival, sprouting, and tubulogenesis. TNC also induces GBM cells to secrete pro-angiogenic factors, such as ephrinB2, a soluble molecule released by MMPs and ADAM10/17 and enhances endothelial cell tubulogenesis. These opposing effects are reminiscent of the cell-specific functions of TNC. Another study suggested that direct contact between the TNC-rich glioma matrix and endothelial cells could induce endothelial detachment, anoikis, selection of a highly proliferative phenotype, and defective tubulogenesis *in vitro*, whereas higher FN : TNC ratios reversed these effects (123). In addition to glioma, TNC also promotes cellular processes involved in angiogenesis in fibrovascular membranes in eyes with proliferative diabetic retinopathy (124) and affects colitis-associated cancer angiogenesis through interaction with integrin αvβ3 (125). Second, TNC can also promote vasculogenic mimicry **(** **Figure 3** **)**. Cai et al. (126) showed that TNC activates the AKT/MMP2/MMP9 axis and further promotes VM in glioma, which is similar to the findings of Kang et al. (127), who reported that *TNC*-knockdown suppresses this process in gastric cancer by inhibiting the ERK-mediated epithelial-to-mesenchymal transition (EMT). The third process involves the mechanism of TNC-induced transdifferentiation of GSCs **(** **Figure 3** **)**. Angel et al. (128) demonstrated the mechanism by which an autocrine TNC–ephrinB2–ephrinB4 signaling pathway supports GSC differentiation into endothelial cells. Taken together, GBM neovascularization correlates with multiple complex processes, in which TNC plays a vital role. In addition to angiogenesis, VM, and transdifferentiation of GSCs, it is necessary to explore additional functions of TNC in co-option or vasculogenesis in future.

### TNC and cell proliferation

**Figure 4:**
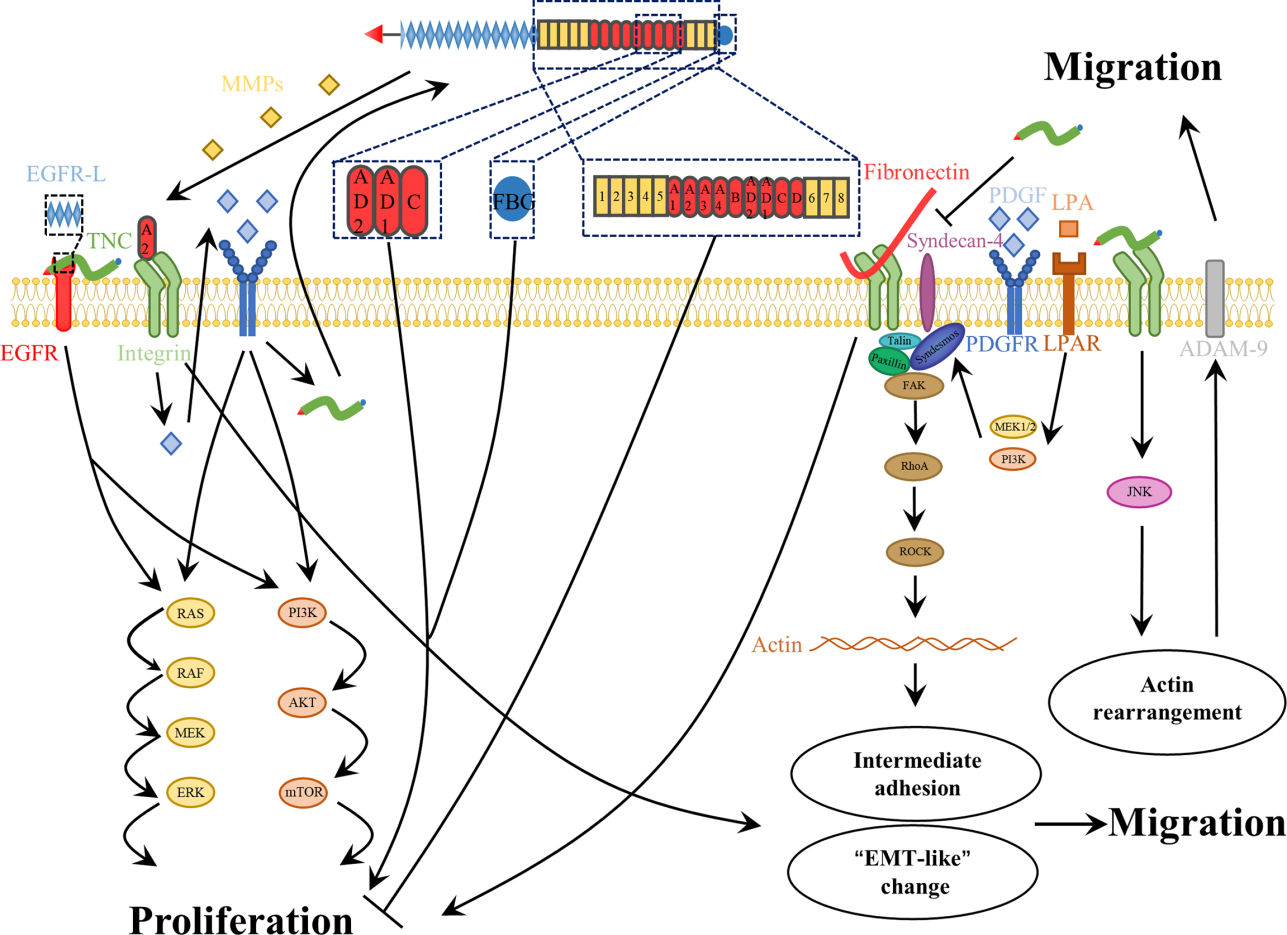
The roles of TNC in glioma cell proliferation and migration. For one thing, the role of TNC in glioma cell proliferation is complex. The EGF-like repeats, the FBG region, A2 and some alternatively spliced domains, such as AD1, -AD2 and -C, of TNC as well as integral TNC molecule contribute to the glioma cell proliferation. In contrast, the fragment composed of all FNIII-domains decreases the proliferation of glioma cell. In addition, TNC impairs the adhesive properties of FN, which contributes to glioma cell proliferation. For another thing, TNC promotes glioma cell invasiveness. This molecule not only contributes to the intermediate adhesion that support cell motility, but also promotes “EMT-like” changes. Moreover, TNC also induces matrix destructing enzymes to promote tumor cell migration.

The TME is composed of heterogeneous cell types, including endothelial, stromal, multiple immune, and tumor cells (25). As an ECM glycoprotein, TNC has been shown to exert different effects on different cells during tumor proliferation. To date, little information has been available on the TNC-mediated signaling pathways involved in cell proliferation. As mentioned above, TNC appears to play a dual role in endothelial cells in glioma (122, 123). Furthermore, the number of microglia in control and TNC-knockdown tumors was not significantly different in xenografts (121). In contrast to microglia, T-cell proliferation can be suppressed by TNC-containing exosomes produced by GSCs (129). Many studies have reported contradictory results regarding the effects of TNC on glioma cells **(** **Figure 4** **)**. Initially, we observed that TNC expression is associated with a high proliferation index (108, 109). Since then, some of the mechanisms underlying the glioma cell proliferation have begun to be revealed. Huang et al. (130) found that TNC was able to bind to FNIII13 in the heparin-binding site II and interfere with cell binding to FN by syndecan-4, thereby leading to human glioma proliferation **(** **Figure 4** **)**. Martin et al. (131) proposed that TNC promotes the growth of tumor cells by inducing the expression of 14-3-3 tau. Differential RNA expression analysis has revealed that some TNC-mediated growth-promoting signaling pathways are activated in glioma cells (132). Other *in vivo* and *in vitro* experiments have also reported the proliferative effects of TNC (128, 133, 134). Moreover, the growth of GSCs is related to TNC-activated NOTCH signaling (87). Nevertheless, the opposite phenomenon was described in another study. TNC failed to affect GBM neurosphere cell growth *in vitro*, whereas *TNC*-knockdown enhanced tumor cell proliferation *in vivo* (121). One possible explanation for these different functions is that different domains of TNC have different proliferative properties (27, 61). The EGF-like repeats and FBG region can bind to the EGF receptor and integrinαvβ3, respectively, contributing to growth. Certain alternatively spliced domains, such as AD1, AD2, and C, are also responsible for proliferation (27, 135). However, a fragment composed of all FNIII domains induced a reduction, whereas the integral TNC molecule led to an increase in glioma cell proliferation **(** **Figure 4** **)** (61). Another explanation is that the absence or decrease of a specific receptor compromises the proliferative effect. The lack of PDGF-Rβ in the glioma cell lines U87, U251, and GL261 resulted in the attenuation of TNIIIA2-related proliferation (136). Recently, Fujita et al. (136–138) showed that TNIIIA2 derived from TNC plays a vital role in the malignant behavior of glioma cells. They presented a positive spiral loop in which the peptide TNIIIA2 promoted PDGF-dependent proliferation by activating integrin β1 in GBM cells expressing PDGF-receptor β. The consequent upregulation of PDGF stimulated TNC expression, which induced MMP-2-mediated TNIIIA2 liberation (136). Further research is needed to explore the relationship between other domains and cell proliferation.

### TNC and cell invasiveness

A major contributor to the poor prognosis of GBM patients is the invasive nature of tumor cells, which readily invade healthy brain tissues. This invasive ability of GBM cells makes maximum safe resection nearly impossible, thereby causing therapy-resistant tumor relapse, even in distant parts of the contralateral hemisphere (139). Generally speaking, glioma cell invasion involves multiple molecular mechanisms, including ECM components, adhesion proteins, proteinases, and cytoskeletal changes (139).

A good balance between assembly and disassembly of cell-matrix adhesion sites is a key determinant of cell adhesion and migration. Appropriate adhesion is essential for migration, yet too strong an adhesion has an adverse effect on cell motility. Hence, intermediate adhesion may be most beneficial for cell migration (140). To date, TNC has been found to play complex modulatory roles in glioma cell adhesion **(** **Figure 4** **)**. Briefly, the dual effects of both pro- and anti-adhesion are dependent on the cell type, cellular context, different receptors, and the structure itself (28, 43, 44). On the one hand, TNC is not a good adhesive substrate for glioma cells, and it can impair fibronectin-mediated cell adhesion and spreading (130). However, some studies have found that TNC acts as a surface-coating molecule that supports cell adhesion (141), perhaps in an Arg-Gly-Asp (RGD)-dependent manner (142). Additionally, adhesion of glioma cells to tenascin is mediated by different coating concentrations and integrin receptors (143). Thus, TNC appears to facilitate regulation of glioma cell adhesion turnover.

Furthermore, many reports have demonstrated that TNC expression is related to the infiltrative phenotype of many tumors, including gliomas (43, 44, 89, 144, 145). Hirata et al. (146) revealed that endogenous TNC enhanced glioma invasiveness through compositional changes in the surrounding brain parenchyma. TNC not only directly supports glioma cell migration, but also augments this role, mediated by FN, through interaction with integrin α2β1 (147). In addition, several mechanisms that are involved in the regulation of TNC expression, such as IL-33/NF-κB/TNC (148) and NOTCH/RBPJκ/TNC (84), were found to increase the motility of glioma cells. Some growth factors, including lysophosphatidic acid and PDGF, strongly induce glioma cell migration *via* actin cytoskeleton remodeling in the TNC microenvironment **(** **Figure 4** **)** (149). Remarkably, although TNC can activate high levels of phosphorylated FAK in endothelial cells, leading to microvascular migration (120), it stimulates low levels of FAK phosphorylation in glioma cells (120, 121). Cell migration is a complex and dynamic process, involving the establishment of polar structures, adhesion formation and disassembly, and formation of protrusions at the front, and contractile structures at the rear of the cell (150). FAK phosphorylation is associated with focal adhesion. Therefore, we speculated that the anti-attachment effect of TNC allows glioma cells to detach from ECM molecules and thereby contribute to migration.

Finally, proteolytic degradation of the ECM is another important cause of glioma invasion. Sarkar et al. used a three-dimensional matrix and revealed that some proteinases are associated with TNC-mediated invasiveness, such as MMP-12 in U178 and U251 glioma cell lines (151, 152), as well as ADAM-9 in glioma patient-derived GSC lines **(** **Figure 4** **)** (153). In addition, *in vivo* experiments also demonstrated its influence on invasion. In TNC-knockdown xenografts, gliomas were confined to well-defined tumor boundaries, and decreased TNC led to inhibition of glioma invasion (121). Considering the diverse actions of distinct domains of this protein, some studies have focused on alternatively spliced domains. TNIIIA2 enhances the disseminative migration of GBM (136, 138) and confers anoikis resistance (137), but these activities are both abrogated by peptide FNIII14, which inactivates β1-integrins (136–138, 154).

Thus, TNC is a potential enhancer of glioma invasiveness. It may be responsible for an intermediate cell adhesion state. Moreover, the aggressiveness of gliomas is further enhanced in the presence of TNC. Further studies involving different domains and isoforms of TNC are required.

### TNC and immunomodulation

**Figure 5:**
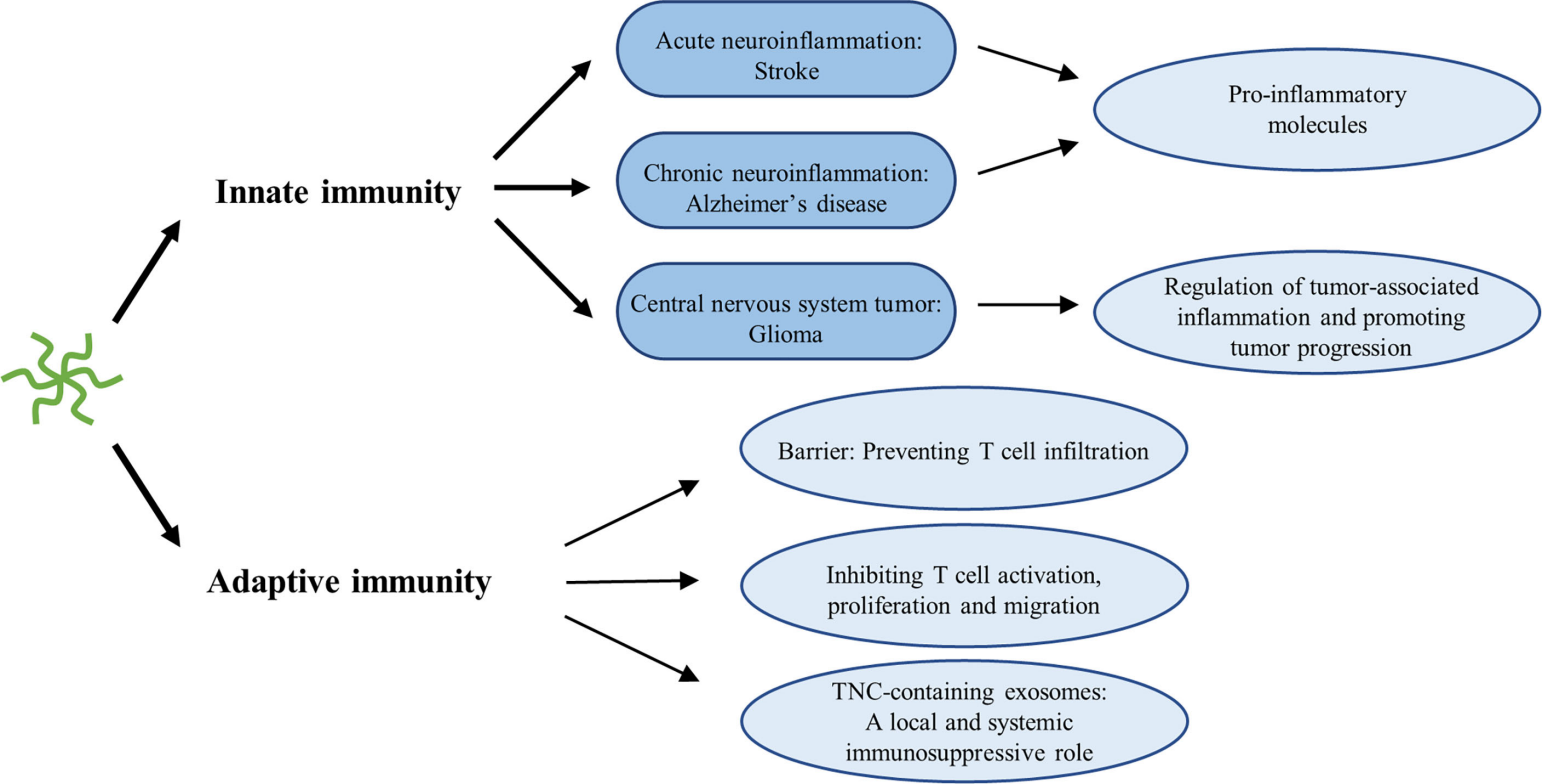
Immunomodulatory role of TNC in neuroinflammation.

Accumulating evidence has indicated that immune cells fail to function properly in gliomas. By employing immune escape mechanisms, including creating an immunosuppressive microenvironment, gliomas can bypass immunosurveillance and hamper the effect of immunotherapy (155). Recently, it has been proposed that serum TNC could be used as an indicator of the immunosuppressive microenvironment status of low-grade gliomas as well as to predict the efficacy of immunotherapy (156). The effect of TNC on various immune cells has been extensively reviewed elsewhere (28, 157). We have focused on the main findings. Generally, TNC activates innate immune cells, but exerts immunosuppressive effects on lymphocytes, such as T cells **(** **Figure 5** **)**. TNC is regarded as an inducer of the neuroinflammatory response in stroke, particularly SAH (56, 157). By interacting with TLR-4 of microglia or macrophages, the FBG domain of TNC leads to the production and release of pro-inflammatory cytokines, including IL-1β, IL-6, and TNF-α (56, 157). In chronic neuroinflammation in the brains of Alzheimer’s disease model mice, TNC deficiency alleviates neuroinflammation and enhances the anti-inflammatory response (69). Hence, it seems reasonable to conclude that TNC has the capacity to induce M1 proinflammatory or anti-tumorigenic phenotypes in macrophages and microglia. However, this phenomenon contrasts with the tumorigenic properties of TNC and its higher expression in high-grade gliomas. One possible explanation is that this dual effect on macrophages is likely to be dependent on the cellular source. In murine models of breast cancer, Deligne et al. (158) found that host-derived TNC enhances antitumor immunity by recruiting proinflammatory macrophages, whereas tumor-derived TNC drives macrophages to produce an immunosuppressive response. Another possibility, proposed by Yalcin et al. (28), is that TNC has different functions in distinct phases of carcinogenesis. TNC plays a pro-inflammatory and anti-tumor role in tumor initiation, and tissue remodeling and tumor-promoting roles during tumor progression. This is similar to the phenomenon in which microglia can inhibit tumor growth and exhibit a tumor-promoting state in the early and late stages of glioma progression, respectively (159). Many studies have illustrated that tumor-associated macrophages (TAMs) are re-educated by glioma cells and show remarkable heterogeneity (160–162). Thus, it is increasingly thought that the M1/M2 dichotomy is an oversimplification and is no longer applicable to gliomas. Due to the complex heterogeneity of TAMs, it is difficult to draw definitive conclusions regarding the role of TNC in TAMs. A study by Ma et al. (86) suggested an intricate relationship between TNC and CD47-mediated macrophage phagocytosis. Decreased CD47, a “don’t eat me” signal, not only recruits more M2-like TAMs, but also upregulates TNC expression, which further facilitates phagocytic ability and secretion of proinflammatory factors. Furthermore, TNC-induced cytokines, such as IL-1β and IL-6, and transcription factors, such as NF-κB and signal transducer and activator of transcription 3 (STAT3), have been implicated in cancer-related inflammation (86, 161, 163). These cytokines or transcription factors also induce TNC expression (7). This positive feedback is consistent with the fact that TNC expression increases with an increase in glioma grade. Hence, a more detailed study may be required to clarify the role of TNC in cancer-related inflammation.

Unlike the innate immunity, the immunosuppressive effects of TNC on T cells have been extensively studied in prostate cancer, breast cancer, and GBM. TNC generates barriers and retains CD8 tumor-infiltrating lymphocytes inside the tumor stroma in human breast cancer (164). TNC expressed on GBM cells decreased the T cell amoeba-like shape formation and paralyzed migration, while transmigration of T cells through the monolayer and ECM of glioma cell lines lacking TNC was obviously increased (165). GSC-derived exosomes carrying TNC attenuate T cell activity by interacting with the integrin receptors α5β1 and αvβ6. More importantly, these TNC-containing exosomes pass through the blood–brain barrier and enter into the circulation, suppressing systemic immune responses in patients with GBM (129). In prostate cancer, Jachetti et al. (166). found that CSCs can migrate early to prostate-draining lymph nodes where they overexpress TNC, inhibiting T cell activation, proliferation, and cytokine production, thereby overcoming immune surveillance. In addition, the relationship between TNC and autophagy was uncovered. TNC has been implicated in the suppression of T-cell antitumor responses caused by autophagy defects. Blockade of TNC sensitizes the efficacy of immune checkpoint inhibitors in autophagy-impaired triple-negative breast cancer (167). This finding may present prospects for anti-PD1/PD-L1 treatment of gliomas.

### Tenascin C and EMT

EMT is an essential process that confers malignant properties to cancer cells (168). Initially, EMT was defined by changes in cell morphology and behavior, such as repression of the existing epithelial characteristics and gain of mesenchymal properties. In recent years, we have found that activation of EMT programs endows cancer cells with additional properties beyond enhanced motility and invasiveness, such as cancer cell stemness, local immunosuppression, increased drug resistance, changes in genomic stability, and prevention of senescence (168). Accumulating evidence has suggested that TNC can promote “EMT-like” changes in different cancers, including breast cancer (169, 170), gastric cancer (127), colorectal cancer (171), pancreatic ductal adenocarcinoma (172), and nasopharyngeal carcinoma (173). However, the EMT observed in gliomas seems to differ from the classical EMT and therefore the term “glial to mesenchymal transition” has been proposed (174). Notably, mesenchymal GBM shares certain common features with gliomas that undergo “EMT-like” changes. They both exhibit a more aggressive nature and resistance to many treatments, and have the same master regulators, such as STAT3 (175–177). Thus, TNC-induced “EMT-like” changes in gliomas require further exploration.

### Tenascin C and mesenchymal GBM

Based on transcriptional signatures, GBMs can be classified into three subtypes: The Cancer Genome Atlas (TCGA) proneural (TCGA-PN), classical (TCGA-CL), and mesenchymal (TCGA-MES) subtypes (178). By applying single-cell RNA-sequencing (scRNA-seq), malignant cells in GBM converge to a limited set of four cellular states: neural-progenitor-like (NPC-like), oligodendrocyte-progenitor-like (OPC-like), astrocyte-like (AC-like), and mesenchymal-like (MES-like) (179). These subtype transitions have become increasingly important, particularly the “proneural-to-mesenchymal transition” (PMT), which is analogous to EMT. This phenomenon is associated with requiring a more aggressive treatment pattern and with resistance to treatment. After reviewing the related literature, we believe that there is an underlying relationship between TNC and MES GBM. Angel et al. (128) found that TNC is overexpressed in MES GBM and that this is positively correlated with the expression of MES markers in TCGA. They further confirmed the role of TNC in regulating glioma cell plasticity in MES GBM. Moreover, Miroshnikova et al. (180) demonstrated that hypoxia-inducible factor 1α (HIF1α) directly regulates TNC expression and that TNC modifies ECM stiffness and mechano-signaling. Interestingly, another study implied that stiff tenascin-rich stroma enhances integrin mechano-signaling to induce PMT in GBMs (181). Additionally, macrophages were found to interact with receptors (OSMR or LIFR) complexed with GP130 on GBM cells *via* macrophage-derived oncostatin M (OSM), thereby activating STAT3 and inducing the transition of GBM cells into MES-like states (182). As mentioned above, TNC is considered an activator of STAT3 (86, 161, 163). Hence, it may be worthwhile to study the significance of TNC in mesenchymal GBM further.

### Tenascin C and treatment-related changes

The current treatment of glioma, particularly GBM, remains a challenging problem with poor prognosis. Although many therapeutic options are available, resistance to therapy frequently causes treatment failure and tumor recurrence. Several studies have shown that TNC are likely to be related to this process. Radiation therapy is known to cause tumor cell death triggered by DNA breaks. However, ionizing radiation also induces changes in the TME, leading to an increase in TNC (183). In addition, irradiation-associated inflammation and hypoxia trigger tenascin C expression *via* TGF-β (80) and HIF-1 signaling (180), respectively. Therefore, it is plausible that radiation therapy can kill tumor cells, while simultaneously providing favorable conditions for tumor relapse.

Elevated expression of TNC affects the response of gliomas to chemotherapy with temozolomide (TMZ). In an *in vitro* experiment, *TNC*-knockdown GBM neurospheres were found to be more sensitive to TMZ (121). Another study conducted *in vivo* and *in vitro* showed that peptide FNIII14, which can inhibit the effects of TNIIIA2 through inactivation of β1-integrins, increased the susceptibility of GBM cells to TMZ by suppressing O^6^-methylguanine-DNA methyltransferase (MGMT) expression (138). These findings suggest that targeting TNC may augment the anti-tumor efficacy of TMZ.

Immunotherapy is a novel therapeutic strategy that extends beyond radio- and chemotherapy. However, the immunosuppressive microenvironment limits the application of immunotherapy in gliomas. We previously reviewed the immunosuppressive effects of TNC, which further compromise the efficacy of anti-cancer immunotherapy. Recently, TTFields therapy have shown promising prospects. Nothing is currently known about TNC function in TTFields.

## Tenascin C in neural stem cell niches: implications for gliomagenesis

### Tenascin C and neural stem cells/oligodendrocyte precursor cells

**Figure 6:**
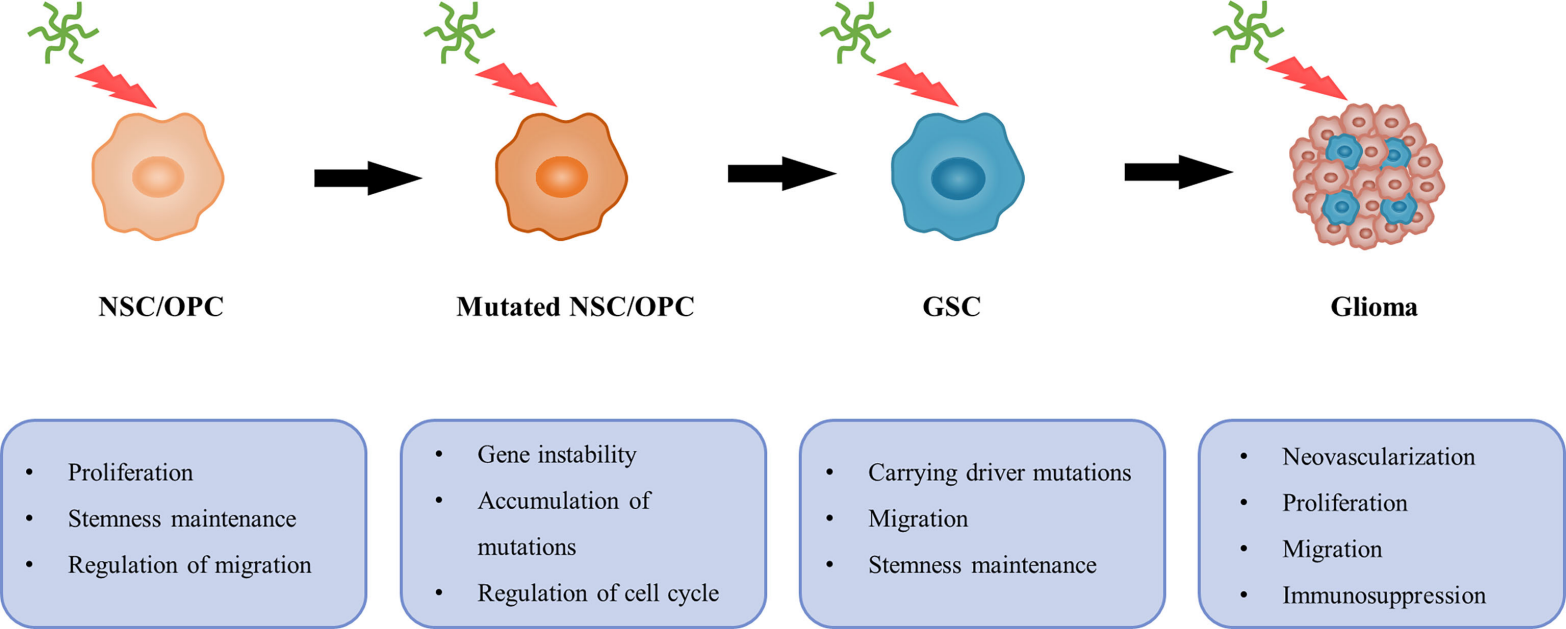
A possible relationship between TNC and gliomagenesis. In general, TNC can promote NSC/OPC proliferation, inhibit differentiation and regulate migration. However, TNC is associated with gene instability and cell cycle, and contributes to accumulation of mutations. Then NSC/OPC carrying driver mutations are likely to undergo GSC transformation. Additionally, neovascularization, proliferation, migration and immunosuppressive effects on T cell also provide favorable conditions, which leads to gliomagenesis.

In the past, neurogenesis was observed to occur mainly during the developmental period. Adult neurogenesis has been widely described. Various newborn neural cells are continuously generated from NSCs located in two canonical regions of the adult CNS: the SVZ of the lateral ventricle and the dentate gyrus of the hippocampus. These stem cells reside in a specialized environment, known as a niche, which is able to maintain the basic properties of stem cells. TNC, secreted by NSCs and OPCs, is considered to be an important component of the stem cell niche and plays a vital role in NSC development (10). The effect of TNC on adult neurogenesis has also been well-characterized (184, 185). Both *in vivo* and *in vitro* assays indicate that *TNC*-knockout reduces OPC proliferation in the mouse CNS, which is associated with a partial loss of response to PDGF (186). In addition, the fact that *TNC*-null OPCs exhibit accelerated maturation rates suggests that this molecule contributes to the maintenance of the immature state (52). The underlying mechanism is gradually being elucidated (187). However, there are distinct perspectives on the role of TNC in OPC migration. Some studies have provided direct evidence for migration-inhibiting effects and mechanisms, including the prevention of WNT signaling (188) and modulation of cell–ECM interactions (189, 190). In contrast, other studies have reported that TNC may be associated with the rostral migratory stream (191), expression of proteinases (151–153) and enhancement of WNT signaling (104, 105). Thus, it is plausible to emphasize the complex migratory role of this molecule. Overall, TNC promotes NSC/OPC proliferation, inhibits differentiation, and regulates migration **(** **Figure 6** **)**.

### Tenascin C and gliomagenesis

Currently, there is a view that SVZ-derived NSCs or OPCs may have potential glioma cellular origins **(** **Figure 6** **)** (30, 192–195). Lee et al. (29) presented direct molecular genetic confirmation from patients and mouse models, showing that NSCs harboring driver mutations in the SVZ migrate to distant brain regions and lead to GBM development *via* aberrant growth of the OPC lineage. Notably, as shown above, TNC is enriched in the GSC and NSC microenvironments and is responsible for tumor progression and neurogenesis, respectively. Additionally, GSCs share many features and behaviors with NSCs, including common markers (such as nestin and CD133) and self-renewal properties (196). Therefore, it is necessary to explore whether TNC is involved in the malignant transformation of NSCs or OPCs.

Previous studies have indicated that malignant gliomas can arise from neural stem/progenitor cells carrying driver mutations (such as mutations in P53, PTEN, EGFR, and NF1), whereas more differentiated cell types are less likely to undergo malignant transformation (29, 30, 195, 197). However, the causes underlying this phenomenon remain unclear. Interestingly, a relationship between TNC and gene instability has previously been reported (198). One RNA profiling experiment showed that TNC downregulates the expression of cell cycle- and DNA repair-related genes in T98G GBM cells (132). The influence of TNC on cell cycle progression of the SVZ and spinal cord neural stem/progenitor cells has been investigated (199, 200). TNC-mediated enhancement of the proliferative capacity of tumor cells (130) and neural stem/progenitor cells (200) concomitantly increases the likelihood of accumulation of mutations. Apart from the above, other important factors, such as stemness maintenance and migration of stem cells, as well as immunosuppressive effects on T cells, also provide favorable conditions for the transformation to malignant tumors. These findings suggest a possible relationship between TNC and gliomagenesis **(** **Figure 6** **)**.

## Clinical significance of tenascin C

The clinical implications of TNC have been extensively recognized **(** **Table 1** **)**. TNC could be a potential prognostic marker for gliomas, including GBM (71, 201), diffuse intrinsic pontine glioma (202), and ependymoma (203). In addition, TNC seems to be regarded as a marker for CSCs (201). Another application is as a candidate for targeted therapy, depending on its characteristic expression pattern. Many ligands targeting TNC have been developed, including F16 (204), G11 (205), R6N (206), PL1 (207), PL3 (208), and Ft (209). The new Ft peptide was synthesized to target glioma-associated TNC and neuropilin-1 synergistically in neovasculature for the specific penetration of nanoparticles in anti-GBM therapy (209). Radiolabeled antibodies specific to distinct domains of TNC have been tested for the treatment of malignant gliomas in clinical studies (210–212). In addition, the TNC aptamer GBI-10 was identified (213) and used to modify adenovirus, thereby improving the adenoviral transduction efficiency in glioma cells (214). In a phase I/II trial, a multi-peptide vaccine, IMA950, that targets TNC held good promise for glioma patients (215). RNA interference has been proposed as a novel strategy for treating gliomas to silence TNC expression. One study by Zukiel et al. (216) suggested that, when double-stranded RNA targeting TNC, known as ATN-RNA, was directly injected postoperatively into the region of resection of 10 patients with glioma, almost all treated patients showed a good response. In agreement with this, other studies have also shown a significant improvement in overall survival without loss of quality of life (217, 218). Magnetic resonance imaging and computed tomography showed delayed tumor growth or a lack of tumor recurrence (217, 218). In future, more rigorous trials will be required to support the clinical application of this treatment.

**Table 1:** The clinical applications of TNC in gliomas.

Categories	Compound	Application	Result	Clinical trial	Ref
**Marker**	TNC	Glioblastoma	Prognosis	—	(71, 201)
	TNC	Diffuse intrinsic pontine glioma	Prognosis	—	(202)
	TNC	Integrated TNC expression and 1q25 status	Prognosis	—	(203)
	TNC	Glioma stem cells	Biomarker	—	(201)
**Antibody**	IL2-F16 (targeting TNC-A1)	Combined with temozolomide	Complete remission	Preclinical	(204)
	G11 (targeting TNC-C)	—	Tumor targeting	Preclinical	(205)
	IL12-R6N (targeting TNC-D)	—	Cancer regression	Preclinical	(206)
**Peptide**	PL1 (targeting FN-EDB and TNC-C) + pro-apoptotic payload	Every other day for 10 total injections	Reduced tumor growth and increased survival	Preclinical	(207)
	PL3 (targeting TNC-C and neuropilin-1) + pro-apoptotic payload	Every other day for 10 total injections	Improved survival	Preclinical	(208)
	Ft peptide (targeting TNC and neuropilin-1) + paclitaxel	Intravenously administered every 2 weeks for 3 times	Improved survival	Preclinical	(209)
**Radiolabeled antibodies**	Radiolabeled anti-TNC monoclonal antibodies such as ^131^I-81C6	Radiotherapy after resection followed by chemotherapy (temozolomide, lomustine, irinotecan, etoposide)	Low toxicity and prolonged survival	Phase I/II	(210–212)
**Aptamer**	GBI-10	GBI-10-modified adenovirus	Improved the adenoviral transduction efficiency	Preclinical	(213, 214)
**Vaccine**	IMA950 (A multi-peptide vaccine IMA950 targeting TNC)	Injections of IMA950/adjuvant poly-ICLC after surgical resection followed by chemoradiotherapy and temozolomide	Improved survival	Phase I/II	(215)
**RNA interference**	ATN-RNA (anti TNC dsRNA)	Injection into the brain after resection	Improved survival	Patients	(216–218)

TNC, Tenascin C; TNC-A1, Tenascin C with extradomain A1; TNC-C, Tenascin C with extradomain C; TNC-D, Tenascin C with extradomain D; FN-EDB, Fibronectin with extradomain B; ds, double-stranded; IL, Interleukin; Ref, References.

## Conclusion

In this review, we focused on the matricellular protein TNC and highlighted its significant implications in gliomas. TNC expression is tightly controlled, with distinct spatial and temporal distribution patterns. Therefore, they are responsible for various physiological and pathophysiological processes. TNC is associated with neurogenesis, as manifested by the promotion of stem cell proliferation, maintenance of stemness, and regulation of migration. In contrast, TNC is implicated in malignant glioma progression, including neovascularization, proliferation, invasiveness, and immunomodulation. However, because of the complex and various domains and the strong crosstalk between them, it is difficult to allocate diverse roles to specific parts of this molecule. Hence, clarifying the underlying mechanisms is a direction for future research. Additionally, based on the hypothesis that SVZ-derived NSCs are instrumental in glioma development, we proposed a possible link between TNC and gliomagenesis, although direct evidence is currently lacking. Therefore, future studies should be conducted to investigate this relationship specifically. Finally, this molecule has promising potential for application in anti-glioma therapy. Many drugs directed against TNC, such as radiolabeled antibodies and dsRNA, have been proven to be effective in preclinical or clinical studies. The selective targeting of the downstream signaling pathways of TNC warrants further investigation. Thorough research on TNC, particularly on the different domains and critical targets of the signaling pathway, will provide new therapeutic strategies for glioma treatment.

## Author contributions

ZF drafted the manuscript and created the figures. GZ, CL, and SS collected the literature. ZD, ZC, YC, and CZ revised the manuscript in English. FL contributed to the correction and final review of this article. All the authors contributed to the study and approved the final version of the manuscript.

## Acknowledgments

We thank all those who participated in the preparation of this manuscript. We would like to thank Editage (www.editage.cn) for English language editing.

## Conflict of interest

The authors declare that the research was conducted in the absence of any commercial or financial relationships that could be construed as potential conflicts of interest.

## Publisher’s note

All claims expressed in this article are solely those of the authors and do not necessarily represent those of their affiliated organizations, or those of the publisher, the editors and the reviewers. Any product that may be evaluated in this article, or claim that may be made by its manufacturer, is not guaranteed or endorsed by the publisher.
